# Supplementary material for: Joint Transcriptome and Metabolome Analysis Prevails the Biological Mechanisms Underlying the Pro-Survival Fight in In Vitro Heat-Stressed Granulosa Cells
Source: Biology (Basel). 2022 May 30;11(6):839. doi: 10.3390/biology11060839 (PMC9220676; doi:10.3390/biology11060839)

Figure S1. Identification of granulosa cells purity, figure taken from previous study [23]. PI was used for staining the cellular nuclei (A,D). The cultured cells were FSHR positive (E). (B) Negative control. (C) is the merged image of (A,B). (F) is the merged image of (D) and (E). Original magnification × 100.

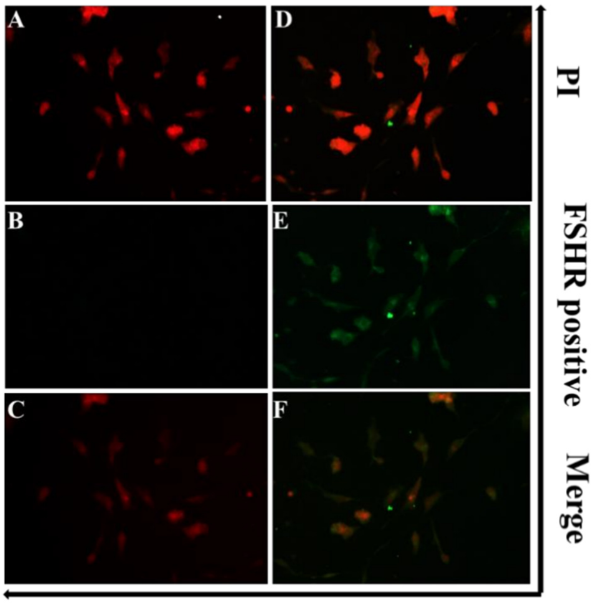

Figure S2. Figure showing the statistical distribution of each of three replicates used for RNA-sequencing and LC-MS based untargeted metabolomics (same samples) of heat-stressed granulosa cells [21,22]. Figure S2A show principle component analysis scores (PCA) plot and distribution dendrogram of transcriptomics study replicates. Figure S2B show partial least square discriminate analysis scores plot (PLSDA) of metabolomics study replicates in negative and positive ion mode of LC-MS analysis.

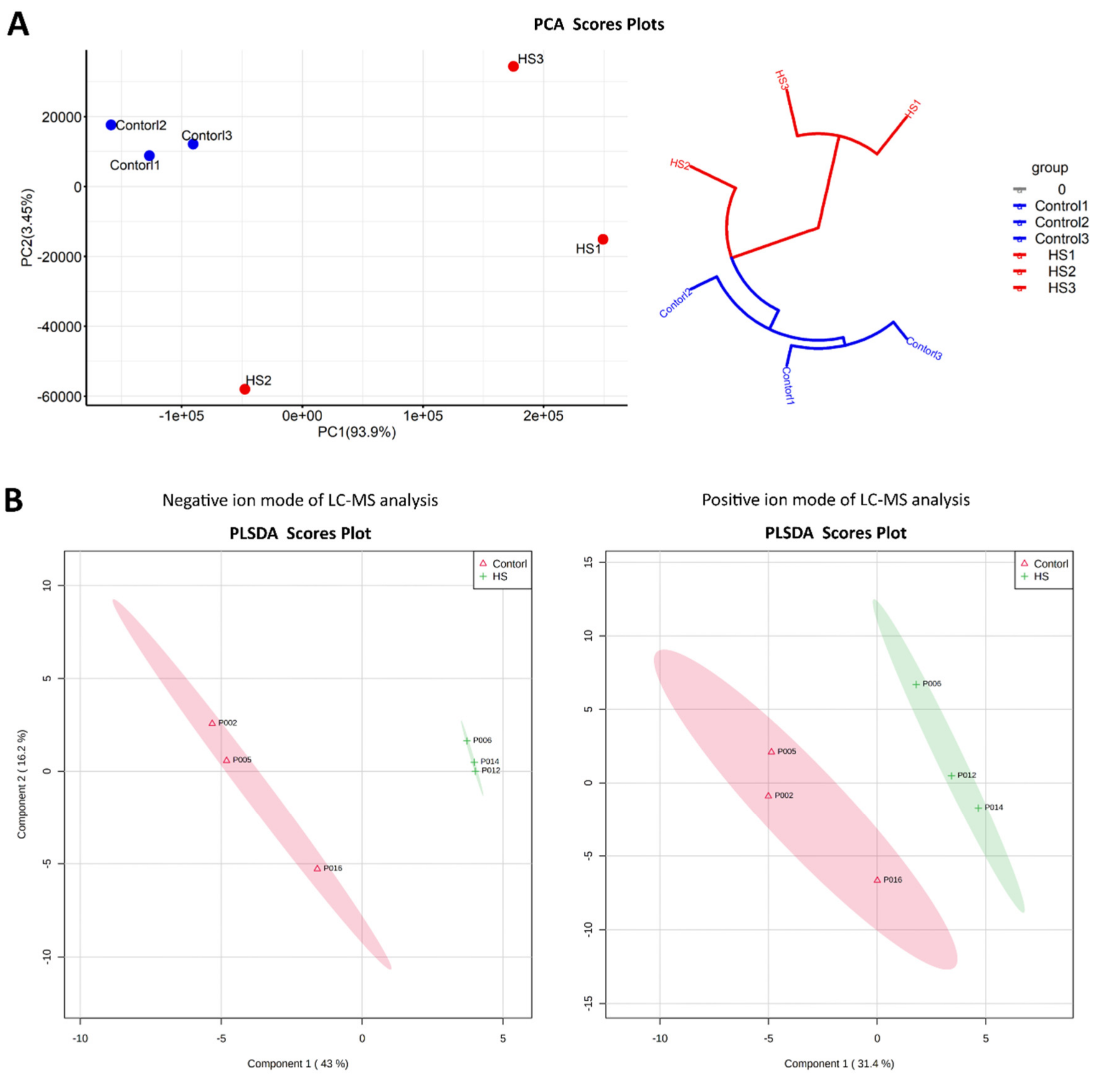

Supplement: Supplementary file 1 [file biology-11-00839-s001.zip › Supplementary Figures S1 and S2.pdf]
